# Prognostic significance of serum NLRC4 in patients with acute supratentorial intracerebral hemorrhage: A prospective longitudinal cohort study

**DOI:** 10.3389/fneur.2023.1125674

**Published:** 2023-03-10

**Authors:** Wei Li, Xuan Lv, Yijun Ma, Yong Cai, Suijun Zhu

**Affiliations:** ^1^Department of Neurosurgery, First People's Hospital of Linping District, Hangzhou, China; ^2^Department of Neurosurgery, Linping Campus, The Second Affiliated Hospital of Zhejiang University School of Medicine, Hangzhou, China

**Keywords:** intracerebral hemorrhage, early neurologic deterioration, outcome, prognosis, severity, biomarkers, NLRC4

## Abstract

**Objective:**

Caspase activation and recruitment domain-containing protein 4 (NLRC4) is implicated in neuroinflammation. The aim of the study was to discern the potential ability of serum NLRC4 in assessment of prognosis after intracerebral hemorrhage (ICH).

**Methods:**

In this prospective, observational study, serum NLRC4 levels were quantified in 148 acute supratentorial ICH patients and 148 controls. Severity was evaluated using the National Institutes of Health Stroke Scale (NIHSS) and hematoma volume, and poststroke 6-month functional outcome was estimated according to the modified Rankin Scale (mRS). Early neurologic deterioration (END) and 6-month poor outcome (mRS 3–6) were deemed as the two prognostic parameters. Multivariate models were established for investigating associations, and receiver operating characteristic (ROC) curves were configured to indicate predictive capability.

**Results:**

Patients had substantially higher serum NLRC4 levels than controls (median, 363.2 pg/ml vs. 74.7 pg/ml). Serum NLRC4 levels had independent correlation with NIHSS scores [β, 0.308; 95% confidence interval (CI), 0.088–0.520], hematoma volume (β, 0.527; 95% CI, 0.385–0.675), serum C-reactive protein levels (β, 0.288; 95% CI, 0.109–0.341) and 6-month mRS scores (β, 0.239; 95% CI, 0.100–0.474). Serum NLRC4 levels above 363.2 pg/ml were independently predictive of END (odds ratio, 3.148; 95% CI, 1.278–7.752) and 6-month poor outcome (odds ratio, 2.468; 95% CI, 1.036–5.878). Serum NLRC4 levels significantly distinguished END risk [area under ROC curve (AUC), 0.765; 95% CI, 0.685–0.846] and 6-month poor outcome (AUC, 0.795; 95% CI, 0.721–0.870). In terms of predictive ability for 6-month poor outcome, serum NLRC4 levels combined with NIHSS scores and hematoma volume was superior to NIHSS scores combined with hematoma volume, NIHSS scores and hematoma volume (AUC, 0.913 vs. 0.870, 0.864 and 0.835; all *P* < 0.05). Nomograms were built to reflect prognosis and END risk of combination models, where serum NLRC4, NIHSS scores and hematoma volume were enforced. Calibration curves confirmed stability of combination models.

**Conclusions:**

Markedly raised **s**erum NLRC4 levels following ICH, in close relation to illness severity, are independently associated with poor prognosis. Such results are indicative of the notion that determination of serum NLRC4 may aid in severity assessment and prediction of functional outcome of ICH patients.

## 1. Introduction

Spontaneous intracerebral hemorrhage (ICH) is one of the commonest lethal stroke subtypes (1). Its pathologic mechanisms mainly include hypertensive arteriopathy and cerebral amyloid angiopathy (2). ICH ranks second in order of stroke incidence, but it leads to a highest percentage of stroke mortality (3). Its poor prognosis is not only correlated with extent of initial hemorrhagic brain injury, but also with occurrence of some in-hospital adverse affairs, such as early neurologic deterioration (END) (4). Thus, it is equally paramount to discriminate patients at risk of either poor prognosis or END in clinical work of ICH (5). During recent decades, some biomarkers have been increasingly noted in neuroscience field with respect to prognostic predictive ability in ICH (6).

ICH-induced secondary brain injury is a harmful factor, thereby worsening neurological function and even resulting in death of patients (7). Neuroinflammation plays an essential role in occurrence and development of secondary brain injury (8). The inflammasome, which is identified as a part of the innate immune system, can cleave pro-caspase-1 to form caspase-1, which subsequently induces pro-interleukin-1beta and pro-interleukin-18 to mature, afterwards transforming into interleukin-1beta and interleukin-18, and finally leading to inflammatory reaction (9). Caspase activation and recruitment domain-containing protein 4 (NLRC4) is expressed in brain tissues and can mediate sterile inflammasome activation in microglia and astrocytes (10). NLRC4 inflammasome contributes to acute brain injury *via* involvement in neuroinflammation and its inhibition could obviously attenuate brain injury, subsequently improving neurological function of rats with ICH or ischemic stroke (11, 12). Hence, it is postulated that NLRC4 may be a biomarker of acute brain injury. In this study, we measured serum NLRC4 levels in a cohort of ICH patients and further strived to explore its prognostic role in human ICH.

## 2. Materials and methods

### 2.1. Participant enrollment

In this prospective, observational, cohort study, we consecutively enrolled patients with non-traumatic supratentorial ICH, who were initially admitted for treatment to our hospital between January 2018 to April 2021. Criteria for participation in the current study included age equal to or above 18 years, voluntary consent to participate in this study, definite verification of the first-time stroke, conservative treatment of hematoma, and hospital admission within the first 24 h after symptom development. Exclusion criteria were as follows: secondary ICH, primary intraventricular hemorrhage, oncopathologies of extra-cerebral and cerebral location, and some specific or severe diseases in the medical history, such as moderate-severe head trauma, and severe cardiac hepatic pulmonary or renal dysfunction. Between April 2020 and April 2021, a group of physical examinees were recruited as controls at our hospital. The study complied with the tenets of the Declaration of Helsinki and was implemented with the approval of the ethics committee at our hospital. Written informed consent for participation in the study was obtained from patients' proxies or controls themselves.

### 2.2. Data obtainment

Upon arrival at emergency room, we inquired about patients' demographics, complaints, medical history, and medication history, as well as checked patients' vital signs. All patients underwent neurological examination with a supplementary assessment of the severity of neurological deficit according to the National Institutes of Health Stroke Scale (NIHSS). Immediate head computerized tomography (CT) scans were accomplished and The ICH diagnosis was established based on CT images. Bleeding size was calculated in accordance with 0.5 × A × B × C formula, where A, B, C are the main maximum dimensions of the hemorrhagic focus measured in three projections (13). Supratentorial hematomas included cerebral lobar and deep ones. Subarachnoidal or intraventricular hematoma was determined on the initial head CT images. END was deemed as an increase of ≥4 in the NIHSS score or death within 24 h after hospitalization (14). Patients were followed up until death or the completion of poststroke 6 months and assessment of 6-month neurological functional status was fulfilled utilizing modified Rankin scale (mRS). In terms of mRS score, scores 3–6 indicated a worse outcome (15).

### 2.3. Immune analysis

Venous blood samples, which were collected from patients and controls, were immediately put in 5 ml gel-containing biochemistry tubes, and then were centrifuged within half an hour. Obtained serum was placed in Eppendorf tubes and preserved at −80°C until analysis. Frozen serum samples were thawed, and afterwards serum NLRC4 levels were in duplicate determined using an enzyme-linked immunosorbent assay kit according to the manufacturer's instruction manual (SinoGeneclon Biotech, HangZhou, China). Two results were averaged for final statistical analysis.

### 2.4. Statistical analysis

The data were statistically analyzed using Statistical Package for the Social Sciences 19.0 (SPSS Inc., Chicago, IL, USA), MedCalc 9.6.4.0 (MedCalc Software, Mariakerke, Belgium) and R software (version 3.5.1; https://www.r-project.org). The Kolmogorov-Smirnov test was done to check normality of distribution of quantitative data. Mean (standard deviation) was given for normally-distributed data, while median (25th and 75th percentiles) was reported for those non-normally-distributed data. To compare the two samples, non-normally distributed continuous variables were assessed by the non-parametric Mann-Whitney U test, normally distributed continuous variables were assessed by the independent-sample *t* test, and categorical variables were assessed by the Chi-square tests or Fisher's exact test where appropriate. To compare the multiple samples, the Kruskal-Wallis test was utilized. The interconnection of the parameters was investigated using the Spearman test and then two multivariate linear regression models were built, where serum NLRC4 levels and mRS scores were regarded as the two independent variables. In addition, two binary logistic regression models were established, where END and 6-month poor prognosis were considered as the two independent variables. To investigate the predictive performance, area under receiver operating characteristic curve (AUC) was employed. A cutoff threshold was selected to yield the maximum Youden index. Nomograms and its calibration curve were plotted for analyzing discriminatory efficiency and stability of models. *P* values < 0.05 were designated as statistically significance.

## 3. Results

### 3.1. Participant selection and characteristics

Initially, a total of 198 supratentorial ICH patients fitted inclusion criteria, and then 50 patients were excluded because of secondary ICH (12 cases), primary intraventricular hemorrhage (8 cases), oncopathologies of extra-cerebral and cerebral location (10 cases), some specific or severe diseases in the medical history (15 cases), loss to follow-up (2 cases), unavailable blood samples (2 cases) and incomplete clinical data (1 case). Eventually, 148 patients were investigated for further analysis. Alternatively, 148 controls were recruited. Table 1 shows that age, gender, body mass index, alcohol drinking and cigarette smoking did not significantly differ between patients and controls (all *P* > 0.05).

**Table 1:** Baseline characteristics between controls and patients with acute supratentorial intracerebral hemorrhage.

**Components**	**Controls**	**Patients**	***P* value**
Age (years)	61.2 ± 15.2	60.6 ± 13.0	0.645
Gender (male/female)	78/70	94/54	0.059
BMI (kg/m^2^)	24.7 ± 4.1	25.5 ± 3.5	0.077
Cigarette smoking	50 (33.8%)	54 (36.5%)	0.626
Alcohol drinking	47 (31.8%)	61 (41.2%)	0.091

Data were shown as frequency (proportion), mean ± standard deviation or median (percentiles 25th-75th) as appropriate. The Chi-square test, Fisher exact test, Student's *t*-test or Mann-Whitney test was in use for intergroup comparisons. BMI means body mass index.

Among this cohort of ICH patients, the investigated chronic diseases included hypertension (93 cases), diabetes mellitus (33 cases), hyperlipidemia (46 cases) and coronary heart disease (15 cases); the collected specific medications included statins (33 cases), anticoagulants (10 cases) and antiplatelet agents (21 cases). As regards radiological characteristics, lobar hematoma accounted for 29.1% (43/148) of all supratentorial hemorrhage, 43 patients had intraventricular hematoma and 13 patients had subarachnoid hematoma. In addition, NIHSS scores ranged from 0 to 17 (median, 6; lower-upper quartiles, 4–11) and hematoma ranged from 3 to 55 ml (median, 17 ml; lower-upper quartiles, 12–27 ml). Admission time since symptom onset varied from 0.5 to 24.0 h (median, 10.6 h; percentiles 25th−75th, 7.0–14.7 h).

### 3.2. Serum NLRC4 levels and disease severity

**Figure 1:**
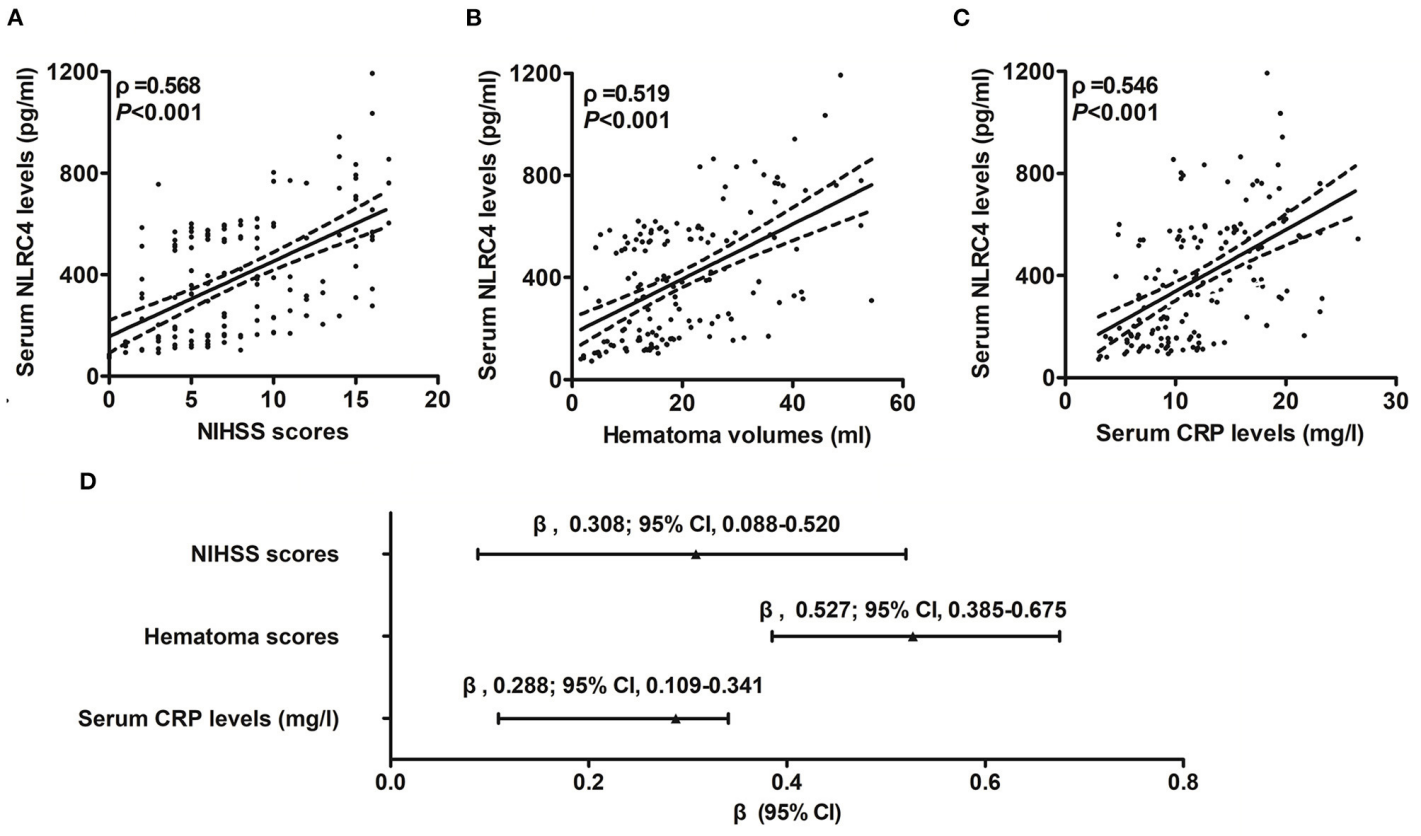
Correlation of serum caspase activation and recruitment domain-containing protein 4 levels with hemorrhagic severity and degree of inflammatory response after acute intracerebral hemorrhage. **(A)** Serum caspase activation and recruitment domain-containing protein 4 levels in correlation with National Institutes of Health Stroke Scale score. **(B)** Serum caspase activation and recruitment domain-containing protein 4 levels in correlation with hematoma volume. **(C)** Serum caspase activation and recruitment domain-containing protein 4 levels in correlation with serum C-reactive protein levels. **(D)** Multivariate results correlated with serum caspase activation and recruitment domain-containing protein 4 levels. Serum caspase activation and recruitment domain-containing protein 4 levels were closely correlated with National Institutes of Health Stroke Scale score, hematoma volume and serum C-reactive protein levels (all *P* < 0.05). NLRC4 indicates caspase activation and recruitment domain-containing protein 4; NIHSS, National Institutes of Health Stroke Scale; 95% CI, 95% confidence interval; CRP, C-reactive protein.

Serum NLRC4 levels of ICH patients ranged from 72.0 to 1,193.5 pg/ml (median, 363.2 pg/ml; percentiles 25th−75th, 163.2–568.4 pg/ml) and those of controls ranged from 10.1 to 319.6 pg/ml (median, 74.7 pg/ml; percentiles 25th−75th, 33.5–95.0 pg/ml). By statistical analysis, ICH patients displayed substantially higher serum NLRC4 levels than controls (*P* < 0.001). In Table 2, besides NIHSS scores (Figure 1A), hematoma volume (Figure 1B) and serum C-reactive protein levels (Figure 1C), intraventricular hemorrhage, blood glucose levels and blood leucocyte count were all highly related to serum NLRC4 levels (all *P* < 0.05). The above-mentioned six significantly correlated variables were incorporated into the multivariate linear regression model and subsequently it was revealed that NIHSS scores, hematoma volume and serum C-reactive protein levels retained to be in independently correlation with serum NLRC4 levels (all *P* < 0.05; Figure 1D).

**Table 2:** Factors in relation to serum caspase activation and recruitment domain-containing protein 4 levels following acute intracerebral hemorrhage.

**Variables**	**ρ**	***P* value**
Age (y)	0.067	0.418
Gender (male/female)	−0.096	0.246
BMI (kg/m^2^)	0.050	0.546
Hypertension	−0.014	0.870
DM	0.015	0.856
Hyperlipidemia	−0.027	0.748
Coronary heart disease	−0.011	0.897
Cigarette smoking	0.008	0.927
Alcohol drinking	−0.054	0.513
Prior usage of statins	−0.007	0.932
Prior usage of anticoagulants	0.100	0.229
Prior usage of antiplatelet agents	0.060	0.468
Admission time (h)	−0.006	0.945
Blood-collection time (h)	−0.043	0.600
SAP (mmHg)	−0.030	0.721
DAP (mmHg)	−0.063	0.450
MAP (mmHg)	−0.037	0.658
Lobar hemorrhage	0.028	0.738
IVH	0.165	0.046
SAH	0.114	0.169
NIHSS scores	0.568	< 0.001
Hematoma volume (ml)	0.519	< 0.001
Serum CRP levels (mg/l)	0.546	< 0.001
Blood leucocyte count (× 109/l)	0.168	0.041
Blood glucose levels (mmol/l)	0.210	0.010

Via the Spearman's correlation coefficient test, correlations were summarized as ρ values. NIHSS denotes National Institutes of Health Stroke Scale; CRP, C-reactive protein; SAH, subarachnoid hemorrhage; IVH, intraventricular hemorrhage; SAP, systolic arterial pressure; DAP, diastolic arterial pressure; MAP, mean arterial pressure; BMI, body mass index; DM, diabetes mellitus.

### 3.3. Serum NLRC4 levels and development of END

**Figure 2:**
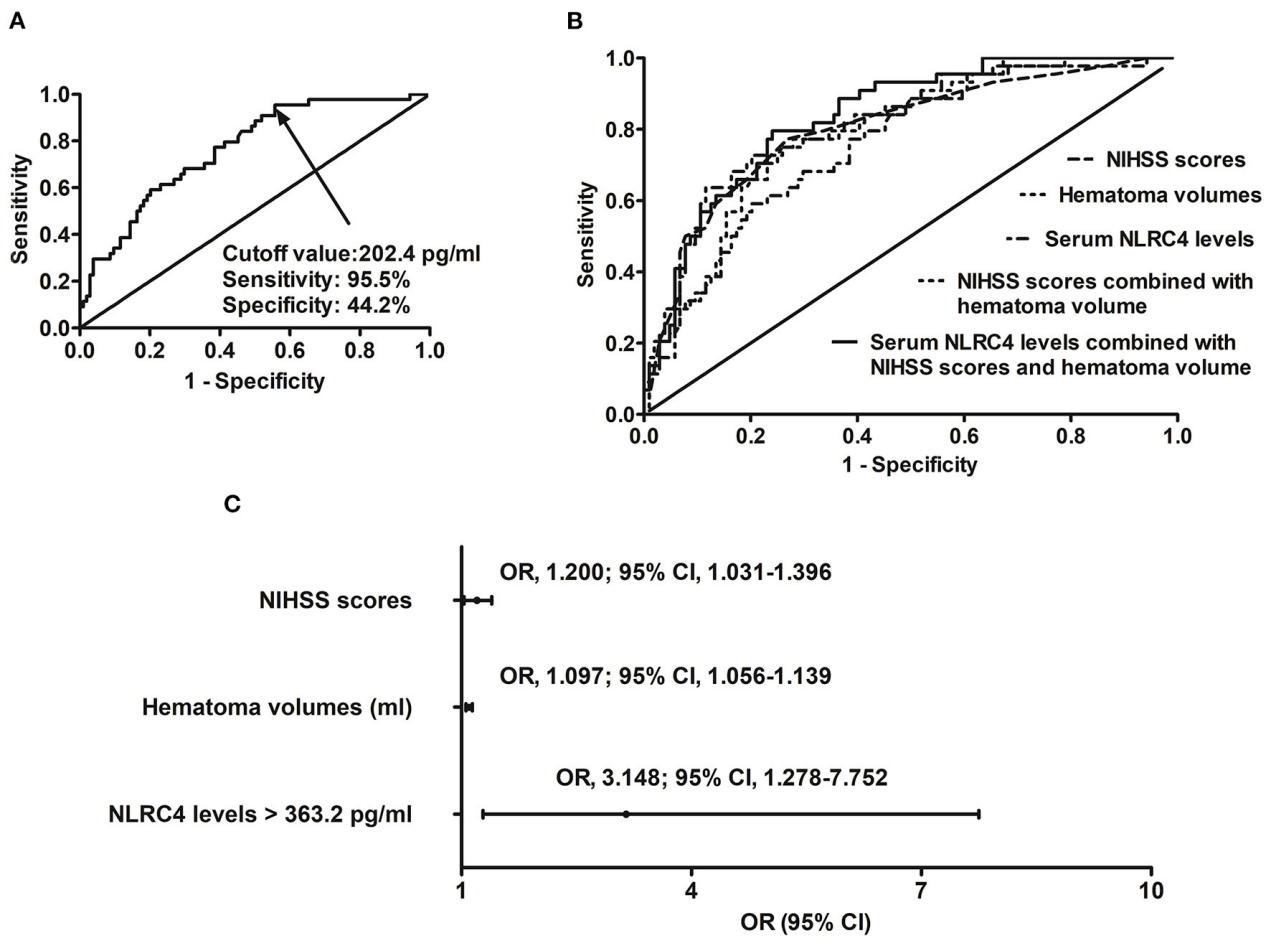
Relationship between serum caspase activation and recruitment domain-containing protein 4 levels and risk of early neurologic deterioration after intracerebral hemorrhage. **(A)** Discriminatory ability concerning serum caspase activation and recruitment domain-containing protein 4 levels for early neurologic deterioration. **(B)** Comparison of distinguishable ability of various variables for early neurologic deterioration. **(C)** Multivariate results associated with early neurologic deterioration. Serum caspase activation and recruitment domain-containing protein 4 levels were independently predictive of early neurologic deterioration and were in possession of high predictive performance for early neurologic deterioration (all *P* < 0.05). NLRC4 indicates caspase activation and recruitment domain-containing protein 4; NIHSS, National Institutes of Health Stroke Scale; 95% CI, 95% confidence interval; OR, odds ratio; END, early neurologic deterioration.

A total of 44 ICH patients experienced END. In patients with development of END, serum NLRC4 levels ranged from 102.6 to 1,193.5 pg/ml (median, 561.2 pg/ml; percentiles 25th−75th, 368.2–748.6 pg/ml); in those with no END risk, they ranged from 72.0 to 854.4 pg/ml (median, 260.4 pg/ml; percentiles 25th−75th, 148.8–523.7 pg/ml). By statistical analysis, END patients exhibited profoundly higher admission serum NLRC4 levels than non-END patients (*P* < 0.001). A cutoff value of serum NLRC4 levels, namely 202.4 pg/ml, was chosen, which predicted END with maximum Youden index (Figure 2A). Under ROC curve (Figure 2B), serum NLRC4 levels (AUC, 0.765; 95% CI, 0.685–0.846) had insignificantly lower predictive ability than NIHSS scores (AUC, 0.802; 95% CI, 0.722–0.882; *P* = 0.464) and hematoma volume (AUC, 0.791; 95% CI, 0.715–0.866; *P* = 0.590), and serum NLRC4 levels combined with NIHSS scores and hematoma volume (AUC, 0.835; 95% CI, 0.769–0.901) displayed insignificantly higher predictive capability than NIHSS scores (*P* = 0.125), hematoma volume (*P* = 0.116), and NIHSS scores combined with hematoma volume (AUC, 0.810; 95% CI, 0.734–0.885; *P* = 0.170).

**Figure 3:**
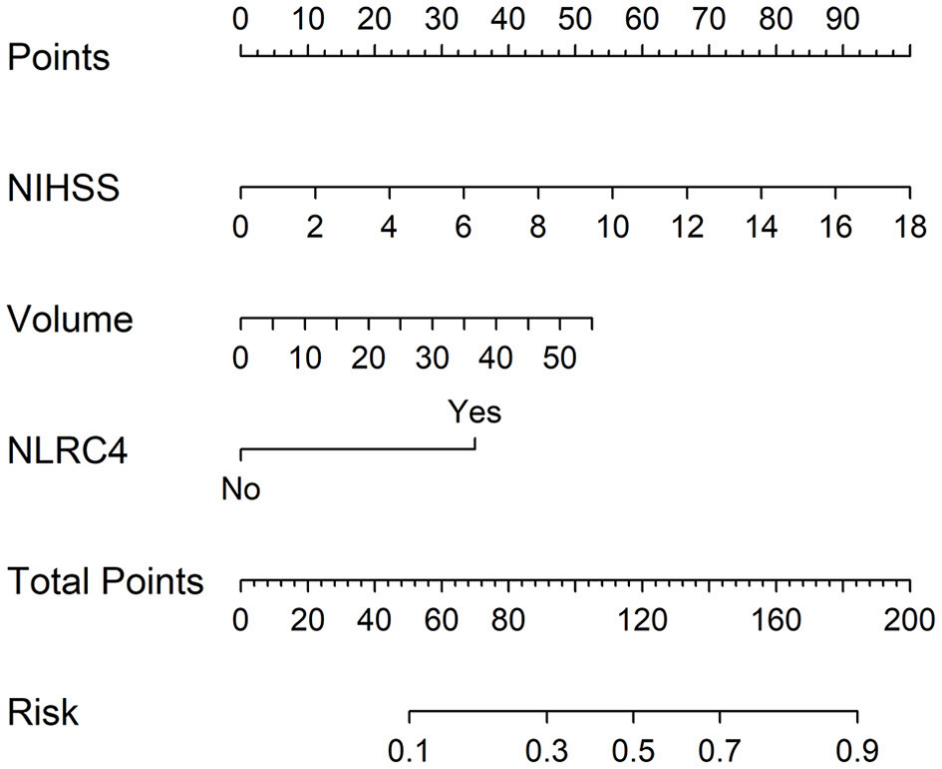
Nomogram for predicting early neurologic deterioration after acute intracerebral hemorrhage. Combination model contained serum caspase activation and recruitment domain-containing protein 4 levels, National Institutes of Health Stroke Scale scores and hematoma volume. Nomogram showed predictive risk assessment. NLRC4 indicates serum caspase activation and recruitment domain-containing protein 4 levels >363.2 pg/ml; NIHSS, National Institutes of Health Stroke Scale; END, early neurologic deterioration.

**Figure 4:**
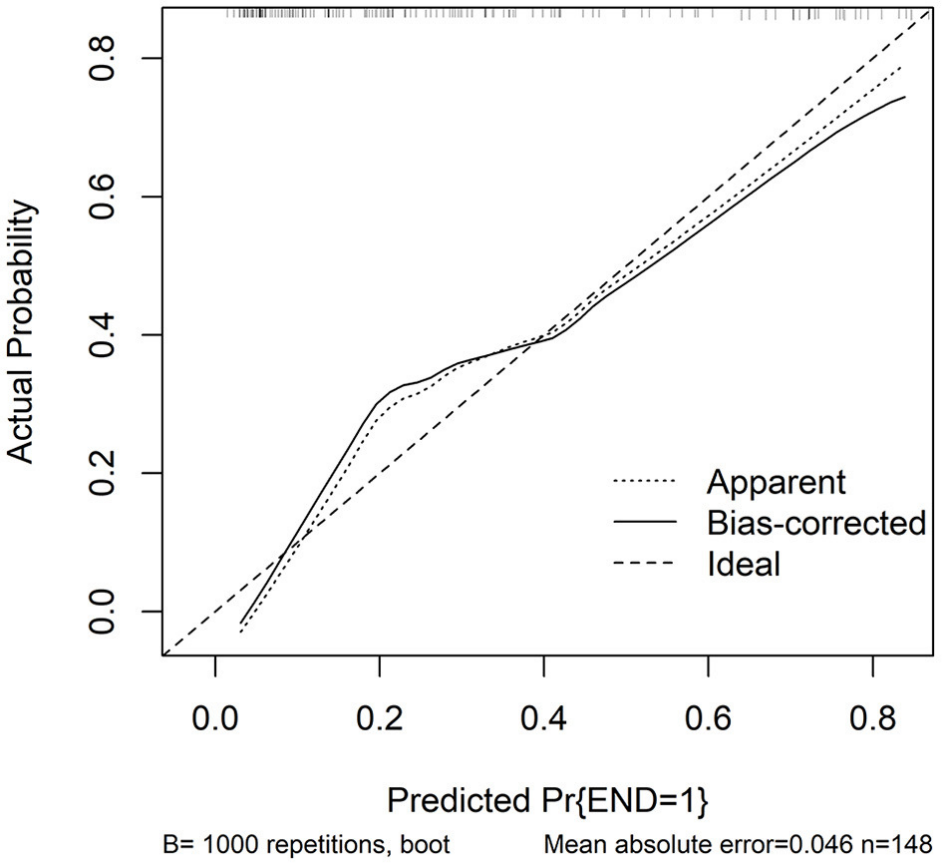
Calibration curve in prediction of early neurologic deterioration. Combination model contained serum caspase activation and recruitment domain-containing protein 4 levels, National Institutes of Health Stroke Scale scores and hematoma volume. Calibration curve displayed that such a combination model had stability to medium-high extent. NLRC4 indicates serum caspase activation and recruitment domain-containing protein 4 levels >363.2 pg/ml; NIHSS, National Institutes of Health Stroke Scale; END, early neurologic deterioration.

In accordance with median value of serum NLRC4 levels (i.e., 363.2 pg/ml), serum NLRC4 levels were considered as the categorical variable. Table 3 displays that, in comparison to patients without development of END, END patients tended to have significantly raised proportions of diabetes mellitus and NLRC4 levels >363.2 pg/ml, and were prone to possess substantially elevated NIHSS scores, hematoma volume, blood glucose levels and serum C-reactive protein levels (all *P* < 0.01). When the preceding significant variables were entered into the binary logistic regression model, NIHSS score, hematoma volume and NLRC4 levels >363.2 pg/ml were the three independent predictors of END (all *P* < 0.05; Figure 2C). In Figure 3, a nomogram, which contained NIHSS scores, hematoma volume and NLRC4 levels >363.2 pg/ml, was established to predict END risk. In Figure 4, its calibration curve showed this model had medium-high stability.

**Table 3:** Factors in relation to early neurologic deterioration following acute intracerebral hemorrhage.

**Variables**	**Presence of END**	**Absence of END**	***P* value**
Age (y)	59.4 ± 13.7	61.1 ± 12.7	0.491
Gender (male/female)	29/15	65/39	0.694
BMI (kg/m^2^)	25.3 ± 3.0	25.5 ± 3.7	0.661
Hypertension	30 (68.2%)	63 (60.6%)	0.382
DM	16 (36.4%)	17 (16.4%)	0.007
Hyperlipidemia	13 (29.6%)	33 (31.7%)	0.793
Coronary heart disease	4 (9.1%)	11 (10.6%)	0.784
Cigarette smoking	18 (40.9%)	36 (34.6%)	0.467
Alcohol drinking	22 (50.0%)	39 (37.5%)	0.158
Prior usage of statins	12 (27.3%)	21 (20.2%)	0.344
Prior usage of anticoagulants	3 (6.8%)	7 (6.7%)	0.985
Prior usage of antiplatelet agents	7 (15.9%)	14 (13.5%)	0.697
Admission time (h)	9.7 (6.3–15.1)	10.9 (7.3–14.7)	0.405
Blood-collection time (h)	11.9 (7.4–16.9)	12.2 (9.0–16.6)	0.554
SAP (mmHg)	141.5 ± 20.9	149.4 ± 25.2	0.070
DAP (mmHg)	83.6 ± 9.4	87.1 ± 11.5	0.074
MAP (mmHg)	102.9 ± 12.6	107.8 ± 15.7	0.064
Lobar hemorrhage	12 (27.3%)	31 (29.8%)	0.756
IVH	14 (31.8%)	27 (26.0%)	0.467
SAH	7 (15.9%)	6 (5.8%)	0.059
NIHSS scores	13 (9–15)	6 (4–9)	< 0.001
Hematoma volume (ml)	27 (19–37)	14 (10–20)	< 0.001
Serum CRP levels (mg/l)	16.6 (11.9–19.2)	10.1 (7.1–13.3)	< 0.001
Blood leucocyte count (× 109/l)	8.6 (6.8–11.3)	8.4 (6.2–10.2)	0.243
Blood glucose levels (mmol/l)	14.0 (10.8–18.0)	11.0 (8.6–13.6)	< 0.001
NLRC4 levels > 363.2 pg/ml	34 (77.3%)	40 (38.5%)	< 0.001

Data were presented as frequency (proportion), mean ± standard deviation or median (upper-lower quartiles). Data were compared using the Chi-square test, Fisher exact test, Student's *t*-test or Mann-Whitney test. NIHSS denotes National Institutes of Health Stroke Scale; CRP, C-reactive protein; NLRC4, caspase activation and recruitment domain-containing protein 4; SAH, subarachnoid hemorrhage; IVH, intraventricular hemorrhage; SAP, systolic arterial pressure; DAP, diastolic arterial pressure; MAP, mean arterial pressure; BMI, body mass index; DM, diabetes mellitus; END, early neurologic deterioration.

### 3.4. Serum NLRC4 levels and experience of 6-month poor prognosis

**Figure 5:**
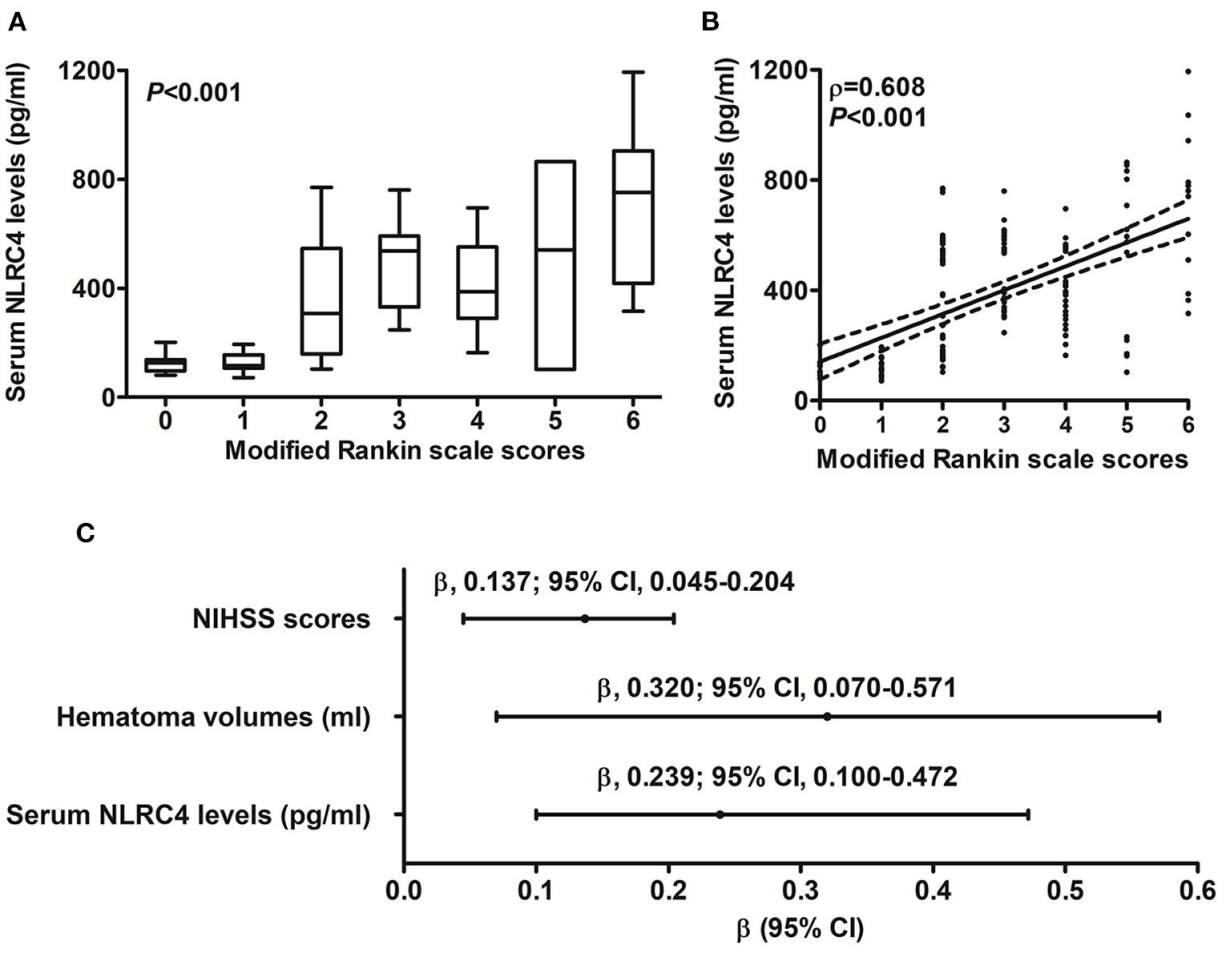
Relationship between serum caspase activation and recruitment domain-containing protein 4 levels and 6-month modified Rankin scale scores after intracerebral hemorrhage. **(A)** Serum caspase activation and recruitment domain-containing protein 4 levels among subgroups divided by modified Rankin scale scores. **(B)** Serum caspase activation and recruitment domain-containing protein 4 levels in correlation to modified Rankin scale scores. **(C)** Multivariate analysis correlated with modified Rankin scale scores. Serum caspase activation and recruitment domain-containing protein 4 levels were strongly correlated with modified Rankin scale scores, which was whether identified as the categorical or continuous variable (all *P* < 0.05). NLRC4 indicates caspase activation and recruitment domain-containing protein 4; NIHSS, National Institutes of Health Stroke Scale; 95% CI, 95% confidence interval.

When serum mRS score was regarded as the categorial variable, serum NRLC4 levels were substantially lowest in patients with mRS score 0, followed by patients with mRS scores 1, 2, 3, 4 and 5 in order of ascending serum NRLC4 levels, and were significantly highest in patients with mRS score 6 (*P* < 0.001; Figure 5A). When serum mRS score was deemed as the continuous variable, serum NRLC4 levels had significantly positively correlation with mRS scores (*P* < 0.001; Figure 5B). In Table 4, besides serum NRLC4 levels, other variables highly correlated with mRS scores referred to diabetes mellitus, intraventricular hemorrhage, NIHSS scores, hematoma volume, serum C-reactive protein levels and blood glucose levels (all *P* < 0.05). The multivariate linear regression model, which contained the aforementioned variables, showed that serum NRLC4 levels, NIHSS scores and hematoma volume were remained independently correlated with mRS scores (all *P* < 0.05; Figure 5C).

**Table 4:** Factors in relation to modified Rankin scale scores at 6 months following acute intracerebral hemorrhage.

**Variables**	**ρ**	***P* value**
Age (y)	0.131	0.113
Gender (male/female)	−0.079	0.339
BMI (kg/m^2^)	−0.072	0.387
Hypertension	0.067	0.415
DM	0.162	0.049
Hyperlipidemia	0.012	0.883
Coronary heart disease	−0.119	0.150
Cigarette smoking	−0.077	0.351
Alcohol drinking	0.074	0.368
Prior usage of statins	0.092	0.268
Prior usage of anticoagulants	0.049	0.554
Prior usage of antiplatelet agents	0.012	0.887
Admission time (h)	−0.105	0.206
Blood-collection time (h)	−0.080	0.331
SAP (mmHg)	0.027	0.746
DAP (mmHg)	0.042	0.609
MAP (mmHg)	0.035	0.670
Lobar hemorrhage	−0.073	0.378
IVH	0.242	0.003
SAH	0.122	0.141
NIHSS scores	0.714	< 0.001
Hematoma volume (ml)	0.657	< 0.001
Serum CRP levels (mg/l)	0.422	< 0.001
Blood leucocyte count (× 109/l)	0.147	0.075
Blood glucose levels (mmol/l)	0.263	0.001
Serum NLRC4 levels (pg/ml)	0.608	< 0.001

Bivariate correlations were performed by the Spearman's correlation coefficient test. NIHSS denotes National Institutes of Health Stroke Scale; CRP, C-reactive protein; NLRC4, caspase activation and recruitment domain-containing protein 4; SAH, subarachnoid hemorrhage; IVH, intraventricular hemorrhage; SAP, systolic arterial pressure; DAP, diastolic arterial pressure; MAP, mean arterial pressure; BMI, body mass index; DM, diabetes mellitus.

**Figure 6:**
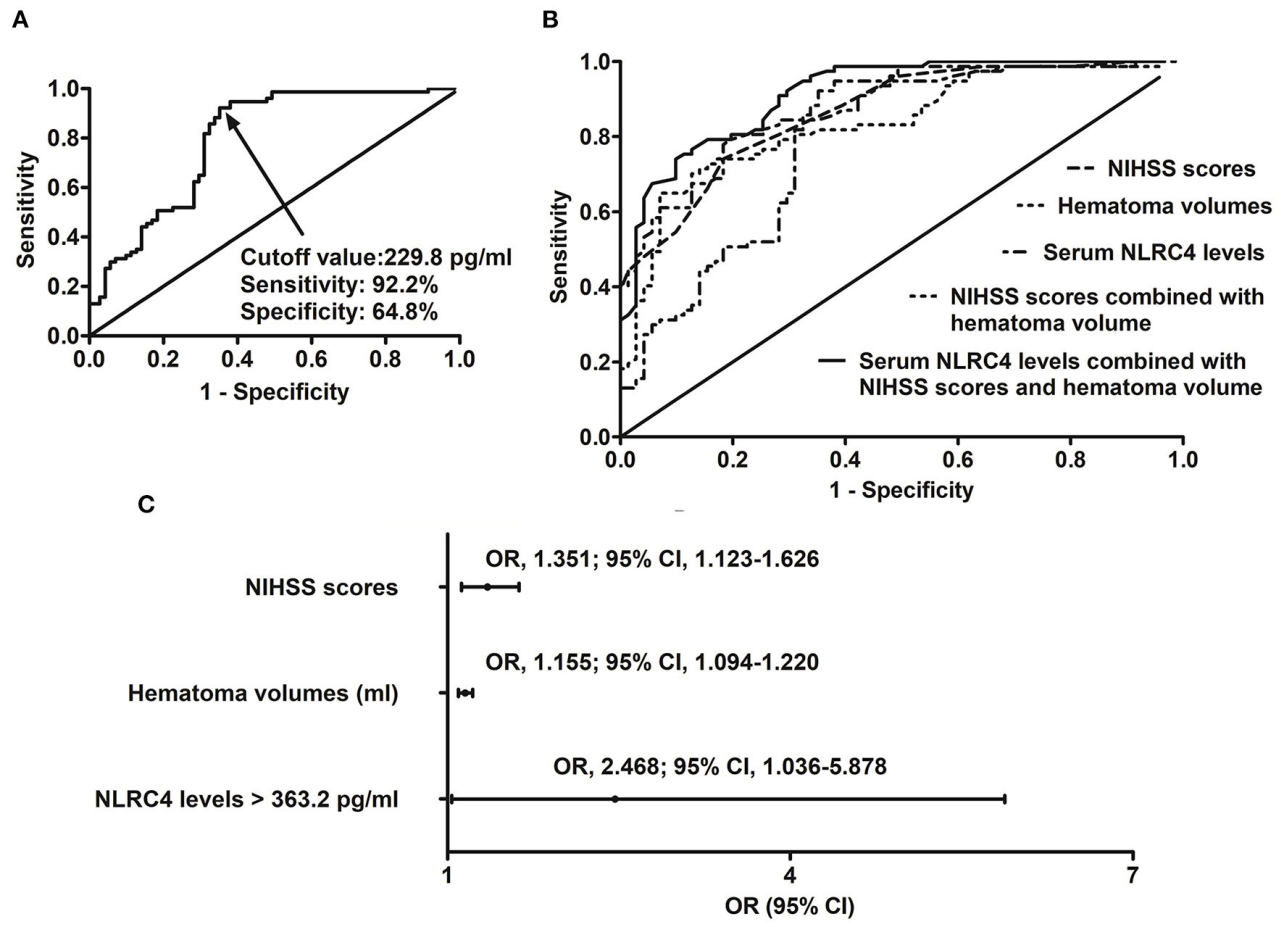
Relation of serum caspase activation and recruitment domain-containing protein 4 levels to poor 6-month prognosis after intracerebral hemorrhage. **(A)** Predictive value of serum caspase activation and recruitment domain-containing protein 4 levels for poor prognosis. **(B)** Comparison of predictive ability of various variables for poor prognosis. **(C)** Multivariate results associated with poor prognosis. Serum caspase activation and recruitment domain-containing protein 4 levels were independently predictive of poor prognosis and were in possession of high predictive power for poor prognosis (all *P* < 0.05). NLRC4 indicates caspase activation and recruitment domain-containing protein 4; NIHSS, National Institutes of Health Stroke Scale; 95% CI, 95% confidence interval; OR, odds ratio.

In patients with possibility of poor prognosis, serum NLRC4 levels ranged from 102.6 to 1,193.5 pg/ml (median, 535.7 pg/ml; percentiles 25th−75th, 324.2–603.8 pg/ml); in those at no risk of poor prognosis, they ranged from 72.0 to 770.8 pg/ml (median, 163.2 pg/ml; percentiles 25th−75th, 124.2–505.6 pg/ml). By statistical analysis, as compared to patients with good prognosis, those with poor prognosis showed substantially enhanced serum NRLC4 levels (*P* < 0.001). Under ROC curve, serum NRLC4 levels >229.8 pg/ml discriminated patients at risk of poor prognosis with maximum Youden index (Figure 6A). In Figure 6B, the prognostic predictive efficiency of serum NRLC4 levels (AUC, 0.795; 95% CI, 0.721–0.870) was equivalent to those of NIHSS scores(AUC, 0.864; 95% CI, 0.808–0.920; *P* = 0.096) and hematoma volume (AUC, 0.835; 95% CI, 0.771–0.900; *P* = 0.349); and serum NRLC4 levels combined with NIHSS scores and hematoma volume (AUC, 0.913; 95% CI, 0.869–0.956) had substantially higher prognostic predictive ability than NIHSS scores (*P* = 0.008), hematoma volume (*P* = 0.002) and combination of NIHSS scores and hematoma volume (AUC, 0.870; 95% CI, 0.816–0.925; *P* = 0.018).

**Figure 7:**
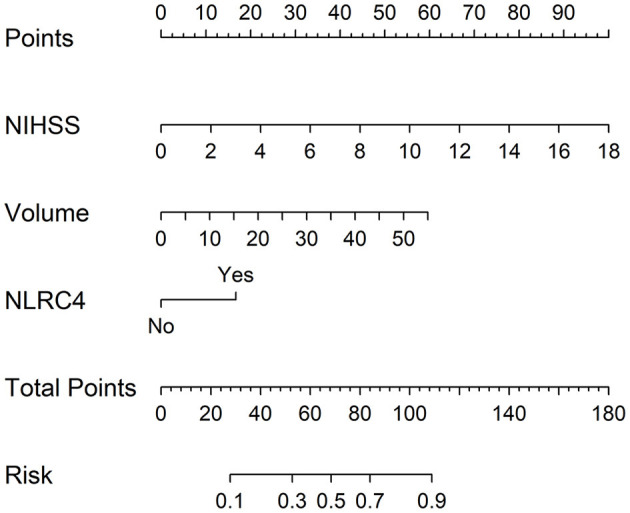
Nomogram for predicting poor 6-month prognosis after acute intracerebral hemorrhage. Combination model contained serum caspase activation and recruitment domain-containing protein 4 levels, National Institutes of Health Stroke Scale scores and hematoma volume. Nomogram showed predictive risk assessment. NLRC4 indicates serum caspase activation and recruitment domain-containing protein 4 levels >363.2 pg/ml; NIHSS, National Institutes of Health Stroke Scale.

**Figure 8:**
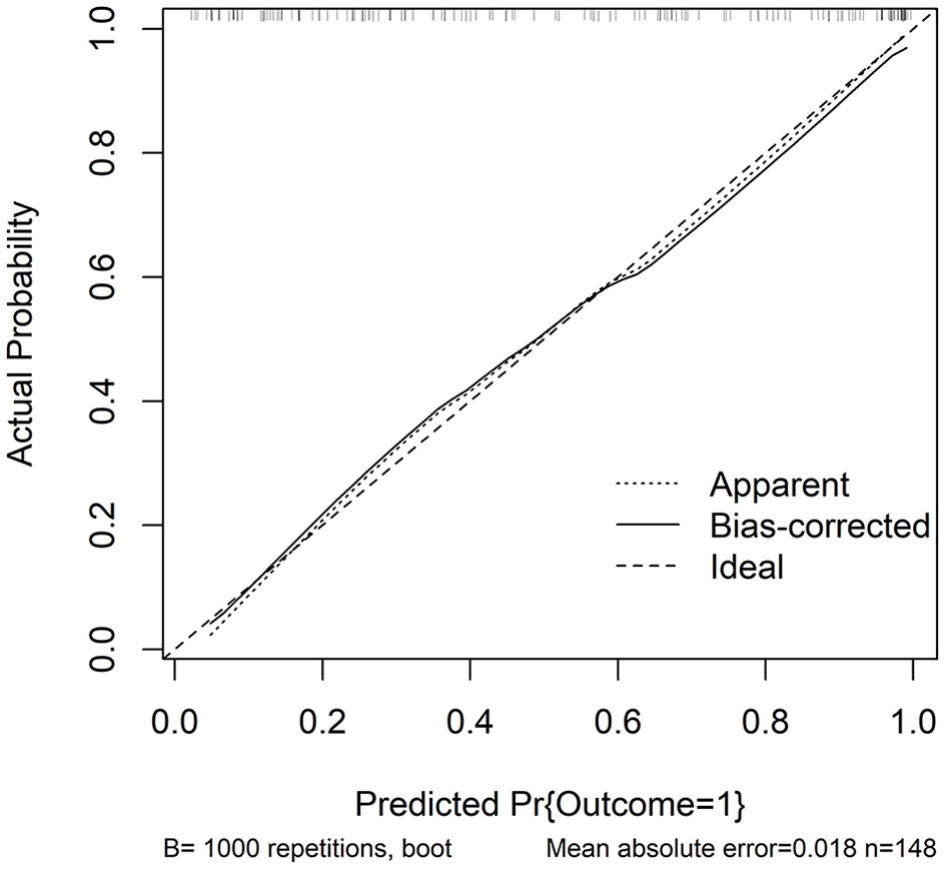
Calibration curve for predicting poor 6-month prognosis after acute intracerebral hemorrhage. Combination model contained serum caspase activation and recruitment domain-containing protein 4 levels, National Institutes of Health Stroke Scale scores and hematoma volume. Calibration curve displayed that such a combination model had stability to medium-high extent. NLRC4 indicates serum caspase activation and recruitment domain-containing protein 4 levels >363.2 pg/ml; NIHSS, National Institutes of Health Stroke Scale.

Just as listed in Table 5, patients with poor prognosis, in comparison to other remainders, were likely to show markedly rising percentage of NLRC4 levels >363.2 pg/ml (*P* < 0.05), and were prone to display substantially increased NIHSS scores, hematoma volume, blood leucocyte count and serum C-reactive protein levels (all *P* < 0.05). The binary logistic regression model, which included the above variables, demonstrated that the factors independently associated with poor prognosis were NIHSS scores, hematoma volume and NLRC4 levels >363.2 pg/ml (all *P* < 0.05; Figure 6C). NIHSS scores, hematoma volume and NLRC4 levels >363.2 pg/ml were combined to differentiate between good prognosis and poor prognosis, whose predictive ability was indicated by a nomogram (Figure 7). In addition, the configured calibration curve confirmed that there is a medium-high stability for such a model (Figure 8).

**Table 5:** Factors in relation to 6-month worse outcome following acute intracerebral hemorrhage.

**Variables**	**mRS 3–6**	**mRS 0–2**	***P* value**
Age (y)	61.7 ± 13.5	59.3 ± 12.4	0.271
Gender (male/female)	45/32	49/22	0.182
BMI (kg/m^2^)	25.1 ± 3.3	25.8 ± 3.8	0.225
Hypertension	51 (66.2%)	42 (59.2%)	0.373
DM	21 (22.3%)	12 (16.9%)	0.130
Hyperlipidemia	26 (33.8%)	20 (28.2%)	0.462
Coronary heart disease	7 (9.1%)	8 (11.3%)	0.661
Cigarette smoking	24 (31.2%)	30 (42.3%)	0.162
Alcohol drinking	38 (49.4%)	23 (32.4%)	0.036
Prior usage of statins	19 (24.7%)	14 (19.7%)	0.469
Prior usage of anticoagulants	7 (9.1%)	3 (4.2%)	0.331
Prior usage of antiplatelet agents	12 (15.6%)	9 (12.7%)	0.612
Admission time (h)	9.8 (6.9–13.8)	11.4 (7.5–15.4)	0.159
Blood-collection time (h)	11.9 (9.1–15.8)	12.9 (8.7–17.5)	0.305
SAP (mmHg)	148.0 ± 24.6	146.0 ± 24.0	0.617
DAP (mmHg)	86.7 ± 11.2	85.3 ± 10.8	0.449
MAP (mmHg)	107.1 ± 15.1	105.5 ± 14.8	0.520
Lobar hemorrhage	24 (31.2%)	19 (26.8%)	0.555
IVH	25 (32.5%)	16 (22.5%)	0.177
SAH	8 (10.4%)	5 (7.0%)	0.472
NIHSS scores	10 (7–15)	4 (3–7)	< 0.001
Hematoma volume (ml)	24 (17–35)	13 (8–16)	< 0.001
Serum CRP levels (mg/l)	13.8 (10.5–18.5)	9.3 (6.7–12.0)	< 0.001
Blood leucocyte count (× 10^9^/l)	9.1 (7.1–11.3)	7.8 (5.9–9.9)	0.015
Blood glucose levels (mmol/l)	12.4 (9.5–15.6)	11.0 (9.3–12.8)	0.115
NLRC4 levels > 363.2 pg/ml	52 (67.5%)	22 (31.0%)	< 0.001

Data were presented as frequency (proportion), mean ± standard deviation or median (upper-lower quartiles). Data were compared using the Chi-square test, Fisher exact test, Student's *t*-test or Mann-Whitney test. NIHSS denotes National Institutes of Health Stroke Scale; CRP, C-reactive protein; NLRC4, caspase activation and recruitment domain-containing protein 4; mRS, modified Rankin scale; SAH, subarachnoid hemorrhage; IVH, intraventricular hemorrhage; SAP, systolic arterial pressure; DAP, diastolic arterial pressure; MAP, mean arterial pressure; BMI, body mass index; DM, diabetes mellitus.

## 4. Discussion

To the best of our knowledge, this is a first series for enrolling a medium sample size of supratentorial ICH patients, and further investigating whether there is a significant elevation of serum NLRC4 levels following ICH, and whether serum NLRC4 acts as an inflammatory biomarker, which is in possession of a prognostic significance in ICH. The main findings of the current study were that (1) serum NLRC4 levels were markedly higher in ICH patients than in controls; (2) serum NLRC4 levels had independent correlation with admission serum C-reactive protein levels, NIHSS scores, hematoma volume and END, as well as 6-month mRS scores and poor prognosis following ICH; and (3) in terms of prognostic predictive ability, combination of serum NLRC4 levels with NIHSS scores and hematoma volume had significantly higher AUC than NIHSS scores, hematoma volume and combination of NIHSS scores with hematoma volume. In a word, determination of serum NLRC4 may be of clinical significance to ICH severity assessment and prognostic prediction.

The pathophysiological processes of acute brain injury caused by ICH are very complicated (1). Besides primary brain injury which is directly induced by hematoma, secondary brain injury is complex, referring to oxidation, neuroinflammation, apoptosis and autophagy (7). Compelling data have demonstrated that secondary brain injury is a crucial factor in the aggravation of neurological dysfunction following ICH (16). Neuroinflammation, an essential participant of secondary brain injury, leads to disruption of the blood-brain barrier and massive neuronal cell death, including apoptosis and necrosis, finally impairing neurological function, and even resulting in death of ICH patients (17).

The nucleotide-binding and oligomerization domain-like receptor family, intracellular innate immune sensors, responds to innate immunity by forming inflammasomes (18). NLRC4 is a member of them and is responsible to process pro-interleukin-1beta and pro-interleukin-18 into a maturation state to promote inflammation (19). A previous experimental study showed that NLRC4 expressions were enhanced in a time-dependent manner following ICH, and peaked at 24 h following ICH (20). Another animal study demonstrated that there was a substantial upregulation of NLRC4 expressions after cerebral ischemia and NLRC4 was mainly localized in neurons (21). NLRC4 could mediate sterile inflammasome activation in microglia and astrocytes (22). Also, NLRC4 substantially enhanced the release of interleukin-1beta and interleukin-18, increased accumulation of microglia, led to neuronal death, damaged blood–brain barrier permeability, and aggravated brain edema of rats with ICH (12). Our study found serum NLRC4 levels were markedly higher in ICH patients than in controls, and moreover elevated NLRC4 levels were independently correlated with serum C-reactive protein levels after ICH. Such findings were supportive of the notion that at least a portion of NLRC4 in peripheral blood of ICH patients may stem from ICH-injured brain tissues, and NLRC4 may be involved in neuroinflammation after ICH. In addition, increased peripheral inflammation, which may be induced *via* neuroimmune interaction, is implicated in pathophysiological processes after ICH and may be highly related to poor prognosis after ICH (23, 24). NLRC4 is responsible to process pro-interleukin-1beta and pro-interleukin-18 into a maturation state to promote inflammation (19). Besides production from cells in central nervous system (21, 22), NLRC4 may be derived from peripheral cells (25, 26). Thus, NLRC4 may participate in inflammatory response after ICH *via* central and peripheral pathways. Moreover, NLRC4 has potential to be a therapeutic target of ICH.

Up to date, it has remained unclear that whether serum NLRC4 levels are correlated with hemorrhagic severity and prognosis after ICH. Using the four multivariate regression models, we revealed the close relationship between serum NLRC4 levels and NIHSS scores, hematoma volume, 6-month mRS scores, END in addition to 6-month poor prognosis after human ICH. More intriguingly, serum NLRC4 levels combined with NIHSS scores and hematoma volume had significantly higher prognostic predictive ability, but not substantially higher END predictive ability, than NIHSS scores, hematoma volume, and combination of NIHSS scores with hematoma volume. Furthermore, the nomogram using combination model was configured to aid in predicting the risk of 6-month poor prognosis and further the built calibration curve showed the stable predictive capability for 6-month poor prognosis after ICH. In consideration of the intimate relation of serum NLRC4 levels to illness severity and long-term poor prognosis, serum NLRC4 may be a promising biochemical marker, which can have the potential to be predictive of worse clinical outcome following acute ICH.

Strengths of our study are that (1) relationships between serum NLRC4 levels and severity plus clinical outcome after ICH were all verified by multivariate analysis; and (2) we only enrolled patients with supratentorial ICH and therefore there is a high homogeneity of data, thereby leading to more scientific and reliable conclusions. Weaknesses of the current study are that (1) a single-center study was undertaken and hence a larger cohort study was warranted to validate the conclusions; (2) although a prospective study was performed to investigate association of serum NLRC4 levels with prognosis, its correlation with serum C-reactive protein levels was determined using only a cross-sectional study; and its effect-cause relationship is still unclear and needs to be further verified; (3) clearly, other proteins related to NLRP1, NLRP3, NLRC4 and AIM2 may be involved in inflammatory role of ICH. This study was designed to investigate prognostic role of serum NLRC4 in ICH. Admittedly, a further study is warranted for determining prognostic roles of other serum proteins in ICH; (4) the aim of this study was to ascertain role of serum NLRC4 as a prognostic biomarker in ICH. Mechanisms pertaining to poor outcome and END may involve its participation in inflammatory reaction. Because some patients would die after admitted into hospital, only admission serum NLRC4 levels can predict risk of poor prognosis and END of all patients. Possibly, to investigating dynamic change of serum NLRC levels would provide some new information; (5) we have made us of calibration curve to validate the stability of such a model. If a separate independent validation is done, it is more perfect.

## 5. Conclusions

To the best of our knowledge, our study, for the first time, found that (1) serum NLRC4 levels, which are statistically significantly elevated after ICH, are correlated independently positively with the intensity of neurological symptoms, hemorrhagic focus volume and extent of peripheral inflammation in ICH patients; (2) serum NLRC4 is an independent predictor of END and 6-month poor prognosis; and (3) when combined with NIHSS scores and hematoma volume, serum NLRC4 is in possession of high prognostic predictive ability. Such data indicate that determination of serum NLRC4, which is identified as an inflammatory biomarker, may be in clinical use for objectification of ICH patients' state and, along with NIHSS scores and hematoma volume, may be allowed to efficiently predict functional outcome after ICH onset.

## Data availability statement

The raw data supporting the conclusions of this article will be made available by the authors, without undue reservation.

## Ethics statement

The studies involving human participants were reviewed and approved by First People's Hospital of Linping District. The patients/participants provided their written informed consent to participate in this study.

## Author contributions

All authors listed have made a substantial, direct, and intellectual contribution to the work and approved it for publication.

## References

[1] Qureshi AI Tuhrim S Broderick JP Batjer HH Hondo H Hanley DF. Spontaneous intracerebral hemorrhage. N Engl J Med. (2001) 344:1450–60. 10.1056/NEJM200105103441907 11346811

[2] Sheth KN. Spontaneous intracerebral hemorrhage. N Engl J Med. (2022) 387:1589–96. 10.1056/NEJMra2201449 36300975

[3] Sansing LH. Intracerebral hemorrhage. Semin Neurol. (2016) 36:223–4. 10.1055/s-0036-1583296 27214696

[4] You S Zheng D Delcourt C Sato S Cao Y Zhang S. Determinants of early versus delayed neurological deterioration in intracerebral hemorrhage. Stroke. (2019) 50:1409–14. 10.1161/STROKEAHA.118.024403 31136288

[5] Law ZK Dineen R England TJ Cala L Mistri AK Appleton JP. Predictors and outcomes of neurological deterioration in intracerebral hemorrhage: results from the TICH-2 randomized controlled trial. Transl Stroke Res. (2021) 12:275–83. 10.1007/s12975-020-00845-6 32902808 PMC7925446

[6] Müller M Tapia-Perez JH Yildiz C Rashidi A Luchtmann M. Alterations in inflammatory markers and clinical outcome after spontaneous intracerebral hemorrhage—preliminary results. J Stroke Cerebrovasc Dis. (2020) 29:104861. 10.1016/j.jstrokecerebrovasdis.2020.104861 32430234

[7] Zhu H Wang Z Yu J Yang X He F Liu Z. Role and mechanisms of cytokines in the secondary brain injury after intracerebral hemorrhage. Prog Neurobiol. (2019) 178:101610. 10.1016/j.pneurobio.2019.03.003 30923023

[8] Aronowski J Zhao X. Molecular pathophysiology of cerebral hemorrhage: secondary brain injury. Stroke. (2011) 42:1781–6. 10.1161/STROKEAHA.110.596718 21527759 PMC3123894

[9] Pandey A Shen C Feng S Man SM. Cell biology of inflammasome activation. Trends Cell Biol. (2021) 31:924–39. 10.1016/j.tcb.2021.06.010 34284921

[10] Duncan JA Canna SW. The NLRC4 inflammasome. Immunol Rev. (2018) 281:115–23. 10.1111/imr.12607 29247997 PMC5897049

[11] Poh L Kang SW Baik SH Ng GYQ She DT Balaganapathy P. Evidence that NLRC4 inflammasome mediates apoptotic and pyroptotic microglial death following ischemic stroke. Brain Behav Immun. (2019) 75:34–47. 10.1016/j.bbi.2018.09.001 30195027

[12] Gan H Zhang L Chen H Xiao H Wang L Zhai X. The pivotal role of the NLRC4 inflammasome in neuroinflammation after intracerebral hemorrhage in rats. Exp Mol Med. (2021) 53:1807–18. 10.1038/s12276-021-00702-y 34848837 PMC8639719

[13] Kothari RU Brott T Broderick JP Barsan WG Sauerbeck LR Zuccarello M. The ABCs of measuring intracerebral hemorrhage volumes. Stroke. (1996) 27:1304–5. 10.1161/01.str.27.8.1304 8711791

[14] Specogna AV Turin TC Patten SB Hill MD. Factors associated with early deterioration after spontaneous intracerebral hemorrhage: a systematic review and meta-analysis. PLoS ONE. (2014) 9:e96743. 10.1371/journal.pone.0096743 24809990 PMC4014549

[15] Sreekrishnan A Dearborn JL Greer DM Shi FD Hwang DY Leasure AC. Intracerebral hemorrhage location and functional outcomes of patients: a systematic literature review and meta-analysis. Neurocrit Care. (2016) 25:384–91. 10.1007/s12028-016-0276-4 27160888

[16] Nobleza COS. Intracerebral hemorrhage. Continuum (Minneap Minn). (2021) 27:1246–77. 10.1212/CON.0000000000001018 34618759

[17] Kashif H Shah D Sukumari-Ramesh S. Dysregulation of microRNA and intracerebral hemorrhage: roles in neuroinflammation. Int J Mol Sci. (2021) 22:8115. 10.3390/ijms22158115 34360881 PMC8347974

[18] Leissinger M Kulkarni R Zemans RL Downey GP Jeyaseelan S. Investigating the role of nucleotide-binding oligomerization domain-like receptors in bacterial lung infection. Am J Respir Crit Care Med. (2014) 189:1461–8. 10.1164/rccm.201311-2103PP 24707903 PMC4226013

[19] Sundaram B Kanneganti TD. Advances in understanding activation and function of the NLRC4 inflammasome. Int J Mol Sci. (2021) 22:1048. 10.3390/ijms22031048 33494299 PMC7864484

[20] Jin P Qi D Cui Y Lenahan C Zhang JH Tao X. Aprepitant attenuates NLRC4-dependent neuronal pyroptosis via NK1R/PKCδ pathway in a mouse model of intracerebral hemorrhage. J Neuroinflammation. (2022) 19:198. 10.1186/s12974-022-02558-z 35922848 PMC9351153

[21] Habib P Harms J Zendedel A Beyer C Slowik A. Gonadal hormones E2 and P mitigate cerebral ischemia-induced upregulation of the AIM2 and NLRC4 inflammasomes in rats. Int J Mol Sci. (2020) 21:4795. 10.3390/ijms21134795 32645874 PMC7370209

[22] Freeman L Guo H David CN Brickey WJ Jha S Ting JP. members NLRC4 and NLRP3 mediate sterile inflammasome activation in microglia and astrocytes. J Exp Med. (2017) 214:1351–70. 10.1084/jem.20150237 28404595 PMC5413320

[23] Jiang C Wang Y Hu Q Shou J Zhu L Tian N. Immune changes in peripheral blood and hematoma of patients with intracerebral hemorrhage. FASEB J. (2020) 34:2774–91. 10.1096/fj.201902478R 31912591

[24] Walsh KB Zhang X Zhu X Wohleb E Woo D Lu L. Intracerebral hemorrhage induces inflammatory gene expression in peripheral blood: global transcriptional profiling in intracerebral hemorrhage patients. DNA Cell Biol. (2019) 38:660–9. 10.1089/dna.2018.4550 31120332 PMC6909779

[25] Souza COS Ketelut-Carneiro N Milanezi CM Faccioli LH Gardinassi LG Silva JS. NLRC4 inhibits NLRP3 inflammasome and abrogates effective antifungal CD8+ T cell responses. iScience. (2021) 24:102548. 10.1016/j.isci.2021.102548 34142053 PMC8184506

[26] Fusco WG Duncan JA. Novel aspects of the assembly and activation of inflammasomes with focus on the NLRC4 inflammasome. Int Immunol. (2018) 30:183–93. 10.1093/intimm/dxy009 29617808 PMC5915955

